# Risk factors for poor prognosis in patients with zoster-associated neuralgia who underwent interventional pain management

**DOI:** 10.3389/fnmol.2024.1393219

**Published:** 2024-10-02

**Authors:** Junpeng Yuan, Youjia Yu, Hong Liu, Huichan Xu, Yan Li, Xiaohong Jin

**Affiliations:** ^1^Department of Pain Medicine, The First Affiliated Hospital of Soochow University, Suzhou, China; ^2^Department of Pain Medicine, Suzhou Xiangcheng People's Hospital, Suzhou, China

**Keywords:** risk factors, zoster-associated neuralgia, interventional pain management, poor prognosis, herpes zoster

## Abstract

**Background:**

Zoster-associated neuralgia (ZAN) is recognized as a challenging neuralgia that often leads to poor prognosis in patients receiving interventional pain management. Identifying risk factors early can enable clinicians to develop personalized treatment plans; however, research in this area is limited.

**Methods:**

We retrospectively screened all patients with ZAN who received interventional therapy in the Pain Department of Soochow University First Affiliated Hospital from January 1, 2022 to August 31, 2023. Data on patient demographics, medical history, neutrophil-to-lymphocyte ratio (NLR), clinical scoring, and treatment methods were collected. Interventional therapy included short-term nerve electrical stimulation (st-NES), pulsed radiofrequency (PRF) and radiofrequency thermocoagulation (RF-TC). Patients were categorized into poor prognosis and control groups based on outcomes 3 months post-discharge. Multivariate logistic regression was used to identify risk factors for poor prognosis.

**Results:**

The final analysis included 282 patients. The rate of poor prognosis was 32.6% (92/282). Multivariate logistic regression analysis revealed that age ≥ 65 years (odds ratio, 2.985; 95% confidence interval, 1.449–6.148; *p* = 0.003), disease duration >3 months (odds ratio, 3.135; 95% confidence interval, 1.685–5.832; *p* < 0.001), head and face pain (odds ratio, 3.140; 95% confidence interval, 1.557–6.330; *p* = 0.001), use of immunosuppressants (odds ratio, 2.737; 95% confidence interval, 1.168–6.416; *p* = 0.021), higher NLR (odds ratio, 1.454; 95% confidence interval, 1.233–1.715; *p* < 0.001), PRF (st-NES as reference) (odds ratio, 2.324; 95% confidence interval, 1.116–4.844; *p* = 0.024) and RF-TC (st-NES as reference) (odds ratio, 5.028; 95% confidence interval, 2.139–11.820; *p* < 0.001) were found to be independent risk factors for poor prognosis in patients with ZAN who underwent interventional pain management.

**Conclusion:**

Age ≥ 65 years (odds ratio, 2.985; 95% confidence interval, 1.449–6.148; *p* = 0.003), disease duration >3 months (odds ratio, 3.135; 95% confidence interval, 1.685–5.832; *p* < 0.001), head and face pain (odds ratio, 3.140; 95% confidence interval, 1.557–6.330; *p* = 0.001), immunosuppressants use (odds ratio, 2.737; 95% confidence interval, 1.168–6.416; *p* = 0.021), higher NLR (odds ratio, 1.454; 95% confidence interval, 1.233–1.715; *p* < 0.001), PRF (odds ratio, 2.324; 95% confidence interval, 1.116–4.844; *p* = 0.024) and RF-TC (odds ratio, 5.028; 95% confidence interval, 2.139–11.820; *p* < 0.001) were identified as independent risk factors for poor prognosis in patients with ZAN who underwent interventional pain management.

## 1 Introduction

Herpes zoster-associated neuralgia (ZAN) is the most common complication of herpes zoster (HZ) and leads to chronic pain manifestations in up to 30% of affected patients (Kawai et al., 2014). Despite the various available treatments, satisfactory outcomes have not been achieved (Johnson et al., 2015). The severity and persistence of pain, in addition to causing distress, significantly impair patients’ quality of life, daily functions, work capabilities, and mental well-being (Drolet et al., 2010). Epidemiological data shows that globally, approximately one-third of the population will develop herpes zoster during their lifetime, with an estimated incidence of 3–5 cases per 1,000 person-years. The burden of ZAN significantly impacts healthcare resources and costs (Kawai et al., 2014; Harvey et al., 2020; Cebrian-Cuenca et al., 2011). The complexity of ZAN pain management continues to constitute a clinical dilemma that perplexes both healthcare providers and patients alike (Drolet et al., 2010).

The underlying mechanisms of ZAN syndrome, potentially linked to neuroinflammation and neuroplasticity, have yet to be fully understood (Baron and Wasner, 2006; Wood, 2002). Medication serves as the foundational treatment approach, yet the efficacy of pharmacotherapy is generally modest and dose dependent (Finnerup et al., 2021). For patients who exhibit resistance to conventional therapies, a combination of interventional treatments offered additional pain relief (van Wijck et al., 2011; Jang et al., 2015). However, the response to interventional pain management, including neuromodulation and denervation techniques, varies significantly among individuals, and the overall success rates have not been entirely satisfactory (Whitley et al., 2010). Therefore, it is crucial to identify high-risk patients early and develop personalized treatment strategies. Currently, such researches on risk factors for poor prognosis in ZAN patients receiving interventional pain management are lacking.

This study aimed to compile and analyze the incidence and risk factors for poor prognosis in ZAN patients receiving interventional pain management. Thus, we are able to identify high-risk patients early and develop personalized treatment strategies, effectively reducing the incidence of poor prognosis in ZAN patients receiving interventional pain management.

## 2 Materials and methods

### 2.1 Ethics and patients

This observational retrospective case–control study was approved by the Ethics Committee of the First Affiliated Hospital of Soochow University (agreement number: 2023545). Informed consent was waived for this study after a waiver was approved by the ethics committee. Patients with ZAN who underwent interventional therapy at the Department of Pain Medicine, the First Affiliated Hospital of Soochow University, from January 1, 2022 to August 31, 2023 were selected as the research objects. The inclusion criteria for patients were as follows: (1) was at least 18 years old; (2) had any period of herpes zoster-associated pain; and (3) underwent the first instance of inpatient interventional pain management. The exclusion criteria for patients were as follows: (1) had intellectual disability or cognitive impairment; (2) had disease such as other pain disorders that confounded the ZAN assessment; and (3) patients with incomplete data.

### 2.2 Diagnosis

Drawing on the source (Watson et al., 1991), this study categorizes the prognosis of ZAN patients as poor if any of the following three conditions manifest within 3 months post-discharge: (1) moderate-to-severe pain, as indicated by an NRS pain score of 4 or higher on a 0 to 10 scale, where higher scores denote worse pain; (2) nighttime awakenings or difficulty initiating sleep due to pain; and (3) the presence of pain-related depression or anxiety necessitated medication. Furthermore, patients who required a second session or multiple sessions of interventional pain management within 3 months post-discharge were also classified as having a poor prognosis in this study.

### 2.3 Data collection

Researchers meticulously reviewed the electronic medical records on an individual basis to gather patient data, which included age (years), sex, BMI (kg/m^2^), disease duration (months), vaccination, antiviral treatment status, pain score (NRS), prodromal pain, breakthrough pain, allodynia, head and face pain, hypertension, diabetes, tumors, immunosuppressant use, and intervention type (including st-NES, PRF, and RF-TC).

Patient information was obtained by direct interview or telephone follow-up after discharge and recorded in the follow-up record system. Exposure years were defined as the number of years since the initial diagnosis of herpes zoster. Significant familial or personal events, such as severe illness, accidents, or the death of a loved one, were also recorded.

After the data collection, a thorough review of the electronic medical records system and the follow-up records system was conducted to ensure the accuracy of the data, and the finalized dataset was used for analysis.

### 2.4 Evaluation scale

Numerical Rating Scale (NRS): The NRS comprises 11 scores ranging from 0 to 10. The patients used a scale that included 11 numbers from 0 to 10 to describe the intensity of the pain, and the greater the number was, the greater the severity of the pain (Bijur et al., 2003).

### 2.5 Statistical analyses

The sample size was estimated using PASS version 15. All available samples were utilized in this study. SPSS 25.0 (IBM) software was used for data processing and analysis. The Kolmogorov–Smirnov test was used to determine whether continuous data were normally distributed. Continuous variables are expressed as the mean ± standard deviation, and the independent sample *t* test was used to compare normally distributed continuous data. Categorical variables were compared using the *χ*^2^ test. Univariate logistic regression was used to screen risk factors through univariate analysis (*p* < 0.1), which were then subjected to multivariate logistic regression analysis, and *p* < 0.05 was considered to indicate statistical significance. The independent variables in the model were analyzed for collinearity. The discrimination and calibration of the multivariable logistic regression model were evaluated using the C-index and Hosmer-Lemeshow test.

## 3 Results

**Figure 1:**
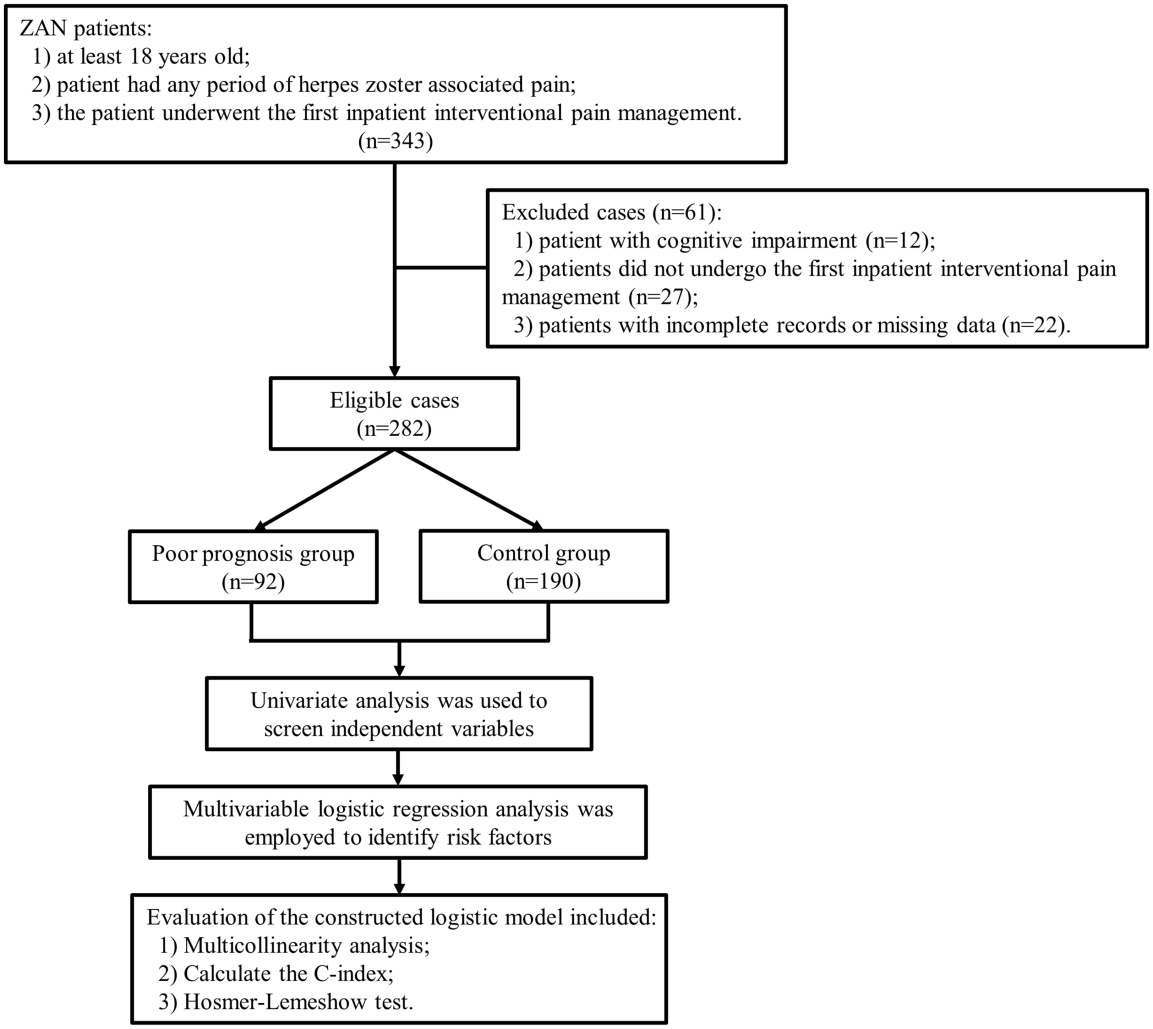
Flow chart.

The final analysis included 282 patients. The poor prognosis group included 92 patients, and the control group included 190 patients, as shown in Figure 1. The rate of poor prognosis was 32.6% (92/282). There was no significant difference in the years of exposure between case patients and controls (*p* = 0.121). No major family or personal events (such as a car accident, serious illness, or death) occurred in either group.

Univariate logistic regression analysis revealed that age ≥ 65 years, disease duration >3 months, head and face pain, diabetes status, immunosuppressant use, NLR, and interventional therapy were the 7 independent variables that needed to be included in the multivariate logistic regression analysis (*p* < 0.1), as shown in Table 1.

**Table 1:** Univariate analysis of risk factors for poor prognosis in patients with ZAN who underwent interventional pain management.

Risk factor	Poor prognosis group (*N* = 92)	Control group (*N* = 190)	Difference or OR (95% Cl)	*p* value
Age, years			2.994 (1.600–5.604) ^b^	<0.001
<65 years	15 (16.3%)	70 (36.8%)		
≥65 years	77 (83.7%)	120 (63.2%)		
Sex			1.070 (0.650–1.761) ^b^	0.791
Female	43 (46.7%)	92 (48.4%)		
Male	49 (53.3%)	98 (51.6%)		
BMI, kg/m^2^	24.9 ± 4.4	24.4 ± 4.2	0.506(−0.568–1.580) ^a^	0.355
Disease duration, months			3.005 (1.783–5.067) ^b^	<0.001
≤3 months	45 (48.9%)	141 (74.2%)		
>3 months	47 (51.1%)	49 (25.8%)		
Vaccination			1.403 (0.484–4.068) ^b^	0.576
No	86	181		
Yes	6	9		
Anti-virus treated			0.883 (0.509–1.534) ^b^	0.659
No	27 (29.3%)	51 (26.8%)		
Yes	65 (70.7%)	139 (73.2%)		
Prodromal pain			1.102 (0.639–1.900) ^b^	0.727
No	64 (69.6%)	136 (71.6%)		
Yes	28 (30.4%)	54 (28.4%)		
Breakthrough pain			1.103 (0.662–1.835) ^b^	0.707
No	55 (59.8%)	118 (62.1%)		
Yes	37 (40.2%)	72 (37.9%)		
Allodynia			0.848 (0.469–1.535) ^b^	0.587
No	22 (23.9%)	40 (21.1%)		
Yes	70 (76.1%)	150 (78.9%)		
Head and face pain			2.070 (1.174–3.650) ^b^	0.011
No	62 (67.4%)	154 (81.1%)		
Yes	30 (32.6%)	36 (18.9%)		
Hypertension			1.130 (0.658–1.939) ^b^	0.658
No	63 (68.5%)	135 (71.1%)		
Yes	29 (31.5%)	55 (28.9%)		
Diabetes			1.952 (0.908–4.196) ^b^	0.083
No	78 (84.8%)	174 (91.6%)		
Yes	14 (15.2%)	16 (8.4%)		
Tumor			1.714 (0.653–4.500) ^b^	0.269
No	84 (91.3%)	180 (94.7%)		
Yes	8 (8.7%)	10 (5.3%)		
Immunosuppressants use			2.830 (1.379–5.809) ^b^	0.003
No	73 (79.3%)	174 (91.6%)		
Yes	19 (20.7%)	16 (8.4%)		
NLR	6.7 ± 1.6	5.4 ± 1.9	−1.286 (−1.750--0.823) ^a^	<0.001
NRS pain score at admission	8.0 ± 1.1	7.8 ± 1.2	−0.168 (−0.459–0.124) ^a^	0.259
Types of intervention			NA	<0.001
st-NES	20 (21.8%)	82 (43.2%)		
PRF	44 (47.8%)	80 (42.1%)		
RF-TC	28 (30.4%)	28 (14.7%)		

OR, odds ratio; BMI, body mass index; NRS, numerical rating scale; NLR, neutrophil-to-lymphocyte ratio; st-NES, short-term nerve electrical stimulation; PRF, pulsed radiofrequency; RF-TC, radiofrequency thermocoagulation.

^aDifference in means.^

^bOR.^

**Figure 2:**
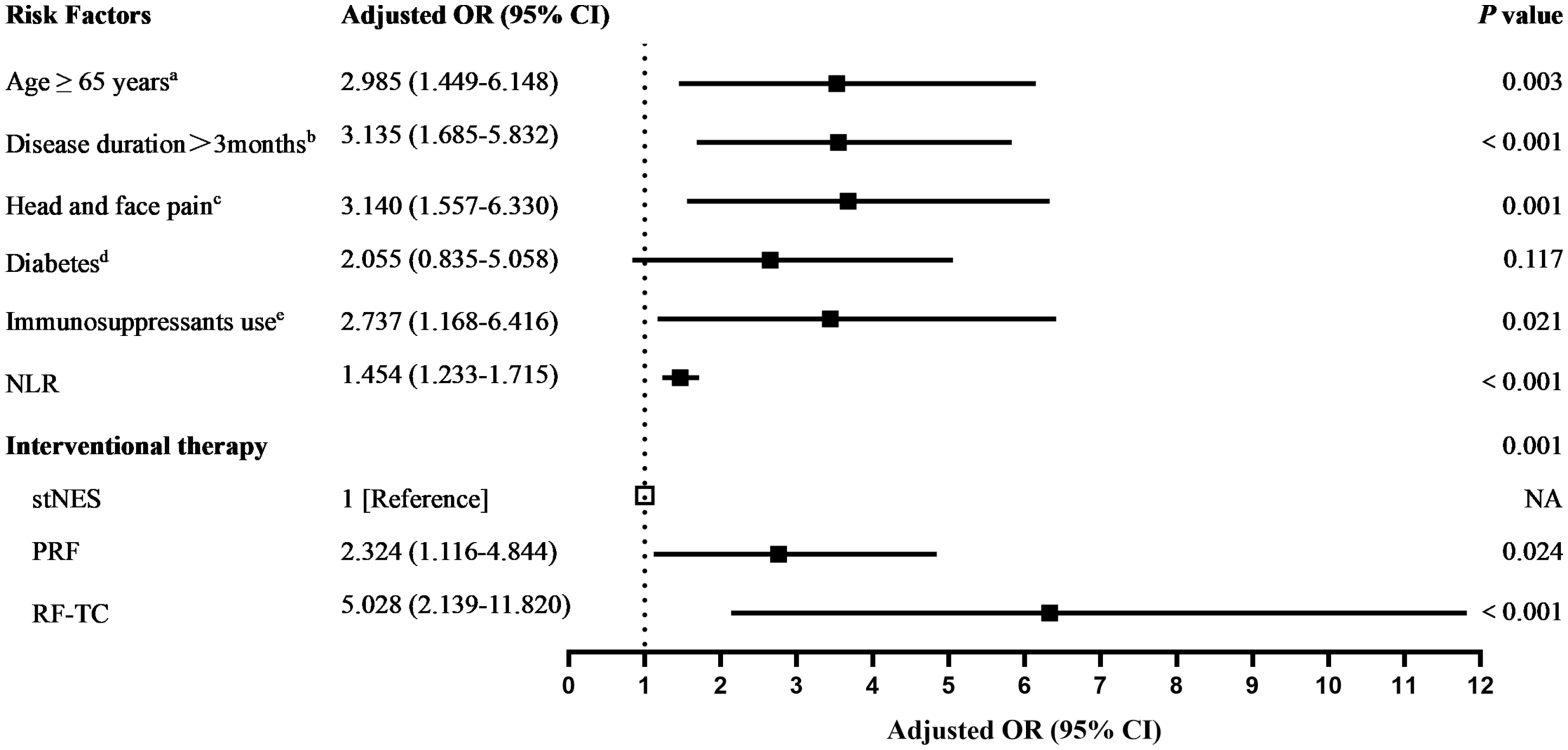
Risk factors for poor prognosis in ZAN patients receiving interventional therapy. NLR, neutrophil-to-lymphocyte ratio; st-NES, short-term nerve electrical stimulation; PRF, pulsed radiofrequency; RF-TC, radiofrequency thermocoagulation. ^a^Patients aged <65 years were used as the reference. ^b^Disease duration <3 months was used as the reference. ^cPatients without head or face pain were used as the reference.^ ^dPatients without diabetes were used as the reference.^ ^ePatients who did not receive immunosuppressive therapy were used as the reference.^

Multivariate logistic regression analysis revealed that age ≥ 65 years (odds ratio, 2.985; 95% confidence interval, 1.449–6.148; *p* = 0.003), disease duration >3 months (odds ratio, 3.135; 95% confidence interval, 1.685–5.832; *p* < 0.001), head and face pain (odds ratio, 3.140; 95% confidence interval, 1.557–6.330; *p* = 0.001), use of immunosuppressants (odds ratio, 2.737; 95% confidence interval, 1.168–6.416; *p* = 0.021), higher NLR (odds ratio, 1.454; 95% confidence interval, 1.233–1.715; *p* < 0.001), PRF (st-NES as reference) (odds ratio, 2.324; 95% confidence interval, 1.116–4.844; *p* = 0.024) and RF-TC (st-NES as reference) (odds ratio, 5.028; 95% confidence interval, 2.139–11.820; *p* < 0.001) were identified as independent risk factors for poor prognosis in patients with ZAN who underwent interventional pain management, as shown in Table 2 and Figure 2.

**Table 2:** Multivariate analysis of risk factors for poor prognosis in ZAN patients who underwent interventional pain management.

Risk factor	Poor prognosis group (*N* = 92)	Control group (*N* = 190)	Adjusted OR	95% CI	*p* value
Age ≥ 65 years^a^	77 (83.7%)	120 (63.2%)	2.985	1.449–6.148	0.003
Disease duration >3 months^b^	47 (51.1%)	49 (25.8%)	3.135	1.685–5.832	<0.001
Head and face pain^c^	30 (32.6%)	36 (19.1%)	3.140	1.557–6.330	0.001
Diabetes^d^	14 (15.2%)	16 (8.4%)	2.055	0.835–5.058	0.117
Immunosuppressants use^e^	19 (20.7%)	16 (8.4%)	2.737	1.168–6.416	0.021
NLR	7.0 ± 1.7	5.3 ± 2.0	1.454	1.233–1.715	<0.001
Interventional therapy					0.001
st-NES	20 (21.8%)	82 (43.2%)	Reference		
PRF	44 (47.8%)	80 (42.1%)	2.324	1.116–4.844	0.024
RF-TC	28 (30.4%)	28 (14.7%)	5.028	2.139–11.820	<0.001

NLR, neutrophil-to-lymphocyte ratio; st-NES, short-term nerve electrical stimulation; PRF, pulsed radiofrequency; RF-TC, radiofrequency thermocoagulation.

^aPatients aged < 65 years were used as the reference.^

^bDisease duration < 3 months was used as the reference.^

^cPatients without head or face pain were used as the reference.^

^dPatients without diabetes were used as the reference.^

^ePatients who did not receive immunosuppressive therapy were used as the reference.^

The constructed multifactorial logistic regression model was subjected to multicollinearity analysis, which indicated that the tolerance values were all greater than 0.1 and that the variance inflation factors (VIFs) were all less than 10, indicating no significant collinearity among the independent variables (Table 3). The C-index was 0.812 with a 95% confidence interval of 0.759–0.866, indicating good discrimination of the model. The Hosmer-Lemeshow test yielded a chi-square value of 8.799 with a *p*-value of 0.360, suggesting a satisfactory calibration of the model.

**Table 3:** Multiple collinearity analysis of independent variables in the multivariate logistic regression model.

Independent variable	Standardization coefficient	*t*	*p* value	Collinearity statistic	
				Tolerance	VIF
Age	0.149	2.881	0.004	0.974	1.027
Disease duration	0.249	5.333	<0.001	0.972	1.029
Immunosuppressants use	0.124	2.384	0.018	0.962	1.039
NLR	0.309	5.913	<0.001	0.957	1.045
Types of intervention	0.183	3.525	<0.001	0.966	1.036
Head and face pain	0.159	3.103	0.002	0.990	1.010

VIF, variance inflation factor; NLR, neutrophil-to-lymphocyte ratio.

## 4 Discussion

In this research, we conducted a multivariate logistic regression analysis of clinical data from 282 patients. These findings indicate that several factors, including age ≥ 65 years, longer disease duration, head and face pain, use of immunosuppressants, higher NLR, PRF (st-NES as reference), and RF-TC (st-NES as reference) are associated with a greater risk of poor prognosis in patients with ZAN who are receiving interventional therapy. This study evaluated the constructed multivariable logistic regression model using the C-index and Hosmer-Lemeshow test, demonstrating that both the discriminative ability and calibration of the predictive model are at a high level. Notably, the NLR is an inflammatory marker and was highlighted for the first time in ZAN treatment. This finding is in accordance with the findings of Schutzer-Weissmann and Farquhar-Smith (2017), who underscore the vital role of inflammatory markers in the management of pain.

The risk factors identified in this study can be categorized into modifiable and non-modifiable factors. Non-modifiable factors, such as age ≥ 65 and head and facial pain, cannot be altered. However, early identification of these high-risk patients is crucial for developing personalized intervention strategies. For such patients, in addition to conventional neuro-modulation therapies, adjunct treatments such as physical therapy can be employed to improve outcomes. Studies suggest that phototherapy may promote scab formation and healing of facial herpes zoster lesions, potentially aiding in facial esthetics recovery (Teitelbaum et al., 2020). However, further large-scale randomized controlled trials are needed to verify the exact efficacy and long-term effect of this neuromodulation therapy combined with physical therapy. On the other hand, modifiable factors, including elevated NLR, the use of immunosuppressants, and early healthcare engagement, offer clear targets for clinical intervention. Optimizing immunosuppressant use and controlling inflammation, along with patient education to encourage early intervention, can significantly reduce the risk of chronic pain.

In our study, 32.6% of patients with ZAN experienced poor outcomes at 3 months post-discharge. Previous research has shown that the rate of poor outcomes following interventional therapy for ZAN varies between 17.2 and 50% (Aggarwal et al., 2020; Isagulyan et al., 2023). The frequency of poor prognosis observed in this study was moderate. In this study, poor prognosis was assessed through a multidimensional approach, adhering to the definition of poor prognosis for ZAN as outlined by Watson et al. (1991). The incorporation of evaluation criteria such as pain score, sleep condition, and emotional state has bolstered the scientific rigor of the outcome endpoint.

This study demonstrated that a higher NLR indicates an increased risk of poor prognosis. The NLR is the ratio of the number of neutrophils to the number of lymphocytes in peripheral blood and is related to the inflammatory state and immune status of the human body (Grivennikov et al., 2010). The NLR has been used to evaluate the outcome of nervous system diseases, and studies have shown that a higher NLR is associated with a poor prognosis in patients with Ramsay Hunt syndrome (Soh et al., 2019). The basic lesions of ZAN are secondary to the inflammatory response of the sensory nervous system and are characterized by neuroinflammatory lesions such as congestion, hemorrhage, inflammatory cell infiltration and demyelination in the ganglion and nerve fibers according to histopathological examination (Gershon et al., 2015). An increase in neutrophils indicates aggravation of the systemic inflammatory response and predicts more severe nerve damage (Bowers et al., 2014). Pain, poor sleep and negative emotions put the body in a high stress state, which causes lymphopenia (Bombeiro et al., 2020; Zhu et al., 2020). An imbalance caused by a strong inflammatory response and immunosuppression is not conducive to viral clearance or neural repair (Amulic et al., 2012). A higher NLR indicates more significant nerve damage and diminished nerve repair capabilities, potentially resulting in heightened pain severity.

This study revealed that pain involving the head and face is a risk factor for poor prognosis, possibly because of the following reasons. First, research indicates that viral invasion of cranial nerves is more likely to lead to persistent pain sequelae (Tsau et al., 2020). Second, HZ infection affecting cranial nerves often co-occurs with complications such as facial paralysis, vestibular dysfunction, eye pain, visual impairment, and vertigo, which can amplify the patient’s feelings of tension, anxiety, and other negative emotions (Liesegang, 2008). Third, facial herpes and hyperpigmentation can further exacerbate anxiety and depression (Tontodonati et al., 2012). Given that the involvement of cranial nerves often leads to significant functional imbalances and facial esthetic concerns, which are difficult to fully recover from even after intervention, patients experiencing these conditions are more susceptible to a poor prognosis.

This study identified age ≥ 65 years, disease duration >3 months, immunosuppressant use, PRF (st-NES as reference) and RF-TC (st-NES as reference) as risk factors for poor prognosis in ZAN patients after interventional therapy. As people age, their immune system gradually weakens, increasing the vulnerability of elderly patients to attacks by the HZ virus, leading to more severe neurological damage (Arvin, 2005). A prolonged disease course can result in sensitization of both the peripheral and central nervous systems, with 3 months considered a critical threshold for sensitization (Fields et al., 1998; Block, 2016). This study revealed that the use of immunosuppressants significantly increases the risk of a poor prognosis. Immunosuppressants diminish the body’s immune response to viruses by inhibiting functional lymphocytes, which also reduces the efficiency of nerve repair (Ehrenstein, 2020). Differences in treatment approaches have led to variations in prognosis. Previous research has demonstrated that short-term nerve electrical stimulation (st-NES) significantly outperforms pulsed radiofrequency (PRF) in treating ZAN (Moriyama, 2009; Liu et al., 2022). RF-TC alleviates pain by destroying nerve fibers, but this can result in numbness and impaired motor function (Mehta et al., 2015). Additionally, a study has indicated that RF-TC might exacerbate pain in nerve fibers that already have pathological neuropathic pain (Chen et al., 2022), suggesting that this destructive treatment should be used with caution.

Both the NLR and the use of immunosuppressants are related to immune status. Our study conducted a collinearity analysis and found no evidence of multicollinearity. This may be due to the wide variety of immunosuppressants used, which can affect neutrophils and lymphocytes differently depending on the stage of the disease, either individually or in combination. The dose–response relationships between different types and doses of immunosuppressants and their effects on neutrophils and lymphocytes also vary.

In this study, the majority of patients were hospitalized for further interventional pain management treatment due to failure of conventional medical treatment. Although our progressive collinearity analysis of all independent variables showed that: There was no significant linear relationship between acute pain and intervention mode, but it is still possible that some patients who experience severe pain in the early stage, without conventional drug treatment, receive interventional treatment. For such patients, early pain and drug treatment may affect the final decision of interventional treatment. PRF and RF-TC may not be considered as independent risk factors for poor prognosis.

The findings of this study offer valuable insights for the personalized management of ZAN. High-risk groups, such as those experiencing head and facial pain or elderly patients, can be identified early, enabling the timely initiation of comprehensive treatment plans, including the use of Chinese medicine treatment or physical therapy (Teitelbaum et al., 2020; Pan et al., 2022). For patients with elevated NLR, early immunomodulation and inflammation control are crucial, while regular follow-up and timely interventions can help reduce the risk of chronic pain. Patients using immunosuppressive agents should have their immune function closely monitored, with medication adjustments made to minimize complications. For those with a prolonged disease course, emphasis should be placed on prevention through increased public awareness and education, helping patients understand the importance of early treatment and timely intervention.

## 5 Limitations

This study has several limitations. Firstly, the retrospective design may introduce information and selection biases. Secondly, being a single-center study, the sample size was relatively small, which limits the generalizability of the findings. Larger, multicenter, prospective studies are needed to validate our results. Thirdly, while research suggests that herpes zoster (HZ) involving the ocular nerves can increase the risk of adverse outcomes (Liesegang, 2008), our limited sample size precluded the inclusion of ocular nerve involvement as a variable. Fourthly, to enhance the study’s rigor, we employed strict screening criteria, which, however, might constrain the external validity of our results. We focused on patients undergoing their first interventional pain treatment to minimize overlap and collinearity among independent variables. Fifth, neural blockade treatment was excluded from our study due to its primary use in outpatient settings and limited effectiveness in treating ZAN (Niv and Maltsman-Tseikhin, 2005).

## 6 Conclusion

Our findings demonstrated that age ≥ 65 years, disease duration >3 months, head and face pain, use of immunosuppressants, higher NLR, PRF (st-NES as reference), and RF-TC (st-NES as reference) were identified as independent risk factors for poor prognosis in patients with ZAN who were receiving interventional pain management.

## Data Availability

The raw data supporting the conclusions of this article will be made available by the authors, without undue reservation.
